# ESOT Guidelines From the Transplantation Learning Journey 3.0

**DOI:** 10.3389/ti.2024.14019

**Published:** 2024-12-17

**Authors:** Umberto Cillo, Ina Jochmans, Nuria Montserrat, Liset H. M. Pengel, Raj Thuraisingham, Nazia Selzner, Annemarie Weissenbacher

**Affiliations:** ^1^ Hepato-Bilio-Pancreatic Surgery and Liver Transplantation Unit, Padova University Hospital, Padova, Italy; ^2^ Transplantation Research Group, Department of Microbiology, Immunology, and Transplantation, KU Leuven, Leuven, Belgium; ^3^ Department of Abdominal Transplant Surgery, University Hospitals Leuven, Leuven, Belgium; ^4^ Pluripotency for Organ Regeneration, Institute for Bioengineering of Catalonia (IBEC), The Barcelona Institute of Science and Technology (BIST), Barcelona, Spain; ^5^ Centro de Investigación Biomédica en Red en Bioingeniería, Biomateriales y Nanomedicina, Barcelona, Spain; ^6^ Institució Catalana de Recerca i Estudis Avançats (ICREA), Barcelona, Spain; ^7^ Erasmus MC Transplant Institute, Erasmus University Medical Center Rotterdam, Rotterdam, Netherlands; ^8^ Department of Renal Medicine and Transplantation, Barts Health NHS Trust, London, United Kingdom; ^9^ Ajmera Transplant Center, University of Toronto, Toronto, ON, Canada; ^10^ Department of Visceral, Transplant and Thoracic Surgery, Center of Operative Medicine, Medical University of Innsbruck, Innsbruck, Austria

**Keywords:** transplantation, clinical practice, consensus platform, guidelines, personalized medicine

We are experiencing an unprecedented transformational era where advancements in personalized medicine are substantially redefining the medical landscape. In this rapidly evolving environment, the future of scientific evidence development and interpretation, along with the effective transmission of clinical guidance, must undergo critical changes.

The scientific community must urgently and proactively facilitate the shift from conventional, “one-size-fits-all” clinical research to more personalized methodologies that thoroughly consider genetic, environmental, and lifestyle factors in a person-centered approach. As this trend accelerates, the methods for generating and interpreting scientific evidence must evolve to address the complexity and granularity of data produced by individualized treatments, ensuring continued relevance in clinical guidance.

In this new context, traditional randomized controlled trials (RCTs), while still valuable, often oversimplify clinical complexities, rendering them inadequate to capture the nuances of personalized interventions. Instead, n-of-1 trials, real-world data, and adaptive trial designs—where individual responses to treatments are closely monitored—are increasingly set to play a central role. This conceptual change is already occurring in areas such as cardiovascular care and oncology, with potentially transformative implications for organ transplantation. The move to individualized care is both essential and urgent in our field, where each patient’s immune system, genetic background, response to immunosuppressive therapy, and multi-procedural history vary widely. Developing scientific evidence that accurately represents this diversity, and reshaping how we translate findings into actionable clinical recommendations, are top priorities. We strongly believe that prominent scientific organizations must embrace the responsibility to promote, guide, and monitor this paradigm shift within their communities.

In line with this, in 2021, the European Society of Organ Transplantation (ESOT) established a taskforce dedicated to guidelines and a platform to activate consensus processes and guideline production within a rigorous methodological environment. Beyond utilizing traditional frameworks for reviewing and evaluating scientific evidence, the ESOT guideline taskforce has prioritized areas within organ transplantation where evidence gaps and/or the transition to precision medicine require expert-driven analysis to inform current clinical guidelines and identify critical research needs for the future. The role of experts in interpreting scientific findings is crucial, as the development of this evidence increasingly incorporates data sources like genomic data, real-world evidence, and adaptive trials. By balancing the promise of personalized care with the rigorous standards of evidence-based medicine, experts serve as critical guides in integrating precision medicine into clinical practice.

To support this historical shift, ESOT has sponsored multiple consensus processes, ensuring robust methodological and logistical support, and created a dedicated platform to facilitate this transition (Cillo et al.).

In this Special Issue, Transplant International publishes the first peer-reviewed articles from this ESOT initiative, offering readers an in-depth exploration of clinical guidance across a range of organ transplantation domains. For example, a consensus led by Park et al. recommends adopting donor-derived cell-free DNA (dd-cfDNA) and urine chemokines (CXCL9 and CXCL10) to identify antibody-mediated rejection in patients experiencing both acute and stable graft dysfunction.

Consensus guidelines led by van den Broek et al. recommend routine, continuous monitoring of donor-specific antibodies (DSA) to optimize long-term kidney graft survival. Although DSA provides valuable insights into subclinical rejection, biopsy confirmation is still necessary for assessing the need for treatment.

Zaza et al. report the first attempt to redefine and standardize pre-implantation biopsy procedures for evaluating kidney grafts from expanded criteria donors (ECD), emphasizing the need for consistent protocols and shared evaluation parameters within the European transplant community.

For the first time, liver transplantation for patients with Primary Sclerosing Cholangitis (PSC) and Inflammatory Bowel Disease (IBD) was addressed in a consensus setting (Carbone et al.). Key challenges—such as the waitlisting process, cancer risks, and heightened perioperative and long-term risks—underline the need for a tailored approach to graft selection, intraoperative management, and postoperative immunosuppression.

Similarly, the first consensus on downstaging, bridging, and immunotherapy in liver transplantation for hepatocellular carcinoma (HCC) patients has been established. Claasen et al. strongly recommend adopting downstaging protocols in HCC patients, regardless of stage, noting that multimodal approaches can significantly improve both recurrence-free and overall survival.

While value-based healthcare and person-centered approaches are now widely recognized as essential to modern medicine, value-based endpoints have yet to be fully developed in organ transplantation. This Special Issue introduces a pioneering consensus on value-based endpoints in liver transplantation, identifying transplant benefit and quality-adjusted life years as the most relevant measures for person-centered outcomes (Carbone et al.). PROMS and PREMS have been identified as important research areas moving forward.

Berenguer et al. conclude that in liver transplantation, biomarkers are still limited in predicting the recurrence of certain liver diseases (e.g., MASH, alcohol relapse, autoimmune diseases). However, these biomarkers show promise in predicting post-transplant HCC recurrence and chronic kidney disease, helping guide clinicians in optimizing immunosuppressive therapies.

In the cardiothoracic setting, Nikolova et al. suggest that peripheral blood gene expression profiling (GEP) assays serve as reliable non-invasive tool to rule out acute cellular rejection in stable, low-risk heart transplant patients. They also indicate that dd-cfDNA measurements could be applied to detect both clinical and subclinical rejection in heart and lung transplants. Emerging biomarkers, including cfDNA epigenetic analysis, fragment omics, exosomes, and microRNA, are currently under investigation.

Ferrer-Fàbrega et al. present an important consensus statement on machine perfusion (MP) in whole pancreas or islet transplantation advocating for a collaborative approach to enhance knowledge evidence in this field.

Amarelli et al. reached broad agreement on the potential of MP technology to expand and improve cardiothoracic organ transplants, recommending the establishment of a pan-European MP registry to promote clinical and cost-effectiveness studies.

Finally, Annema et al. address the often-overlooked topic of prehabilitation for transplant candidates, advocating a multimodal strategy that emphasizes exercise, nutrition, and psychosocial support to improve outcomes. A coordinated effort and a core outcome set for future research are proposed to address the shortage of high-quality studies in this area.

In conclusion, this Special Issue compiles the outcomes of methodologically rigorous consensus processes, balancing existing evidence with expert insights to provide clinical guidance in several critical, previously unexplored areas of organ transplantation. We are confident that readers will find this Special Issue an innovative overview, presenting a broad perspective on precision medicine in organ transplantation and posing significant questions for future research.

